# Unusual Intraventricular Bullet Migration During Operation: A Case Report on Treatment Strategies

**DOI:** 10.7759/cureus.41554

**Published:** 2023-07-08

**Authors:** Fernando De Nigris Vasconcellos, Augusto Müller Fiedler, Pedro Biasi, Matheus Brunet, Leonardo Frighetto, Bernardo A Monaco, Joacir G Cordeiro, Timoteo Almeida

**Affiliations:** 1 Department of Neurosurgery, NYU (New York University) Langone Health, New York, USA; 2 Department of Neurosurgery, University of Miami, Miami, USA; 3 Department of Neurosurgery, Sao Vicente de Paulo Hospital, Passo Fundo, BRA; 4 Department of Neurosurgery, Moinhos de Vento Hospital, Porto Alegre, BRA; 5 Department of Neurosurgery, Clinica de Dor e Funcional, São Paulo, BRA; 6 Department of Neurosurgery, University of São Paulo, São Paulo, BRA; 7 Department of Radiation Oncology, University of Miami, Miami, USA

**Keywords:** intraventricular lodgement, intraventricular bullet migration, traumatic brain injury, gunshot head wound, penetrating brain injury

## Abstract

Gunshot head injuries are increasingly prevalent in the urban setting and carry complex technical and clinical decision-making challenges to practicing neurosurgeons. Here, we present a unique case of a patient who suffered a gunshot injury and presented to the emergency department with an intraventricular bullet lodgment without significant neurological deficits. The patient was rushed to the operating room to remove the bullet after neuroimaging demonstrated its migration inside the ventricular system. The patient showed a favorable outcome postoperatively. This case report highlights the importance of prompt diagnosis and tailored management strategies in cases of intraventricular bullet lodgment.

## Introduction

Penetrating brain injuries often carry a worse prognosis due to the complexity of the injury and associated complications. Gunshot wounds are common and increasingly frequent in the urban setting, resulting in a wide range of complex intracranial pathologies [1]. Among these, intraventricular lodgment and migration within the ventricular system are scarcely documented phenomena in the literature. Understanding such cases' unique characteristics and management considerations is crucial for optimizing patient outcomes.

## Case presentation

**Figure 1:**
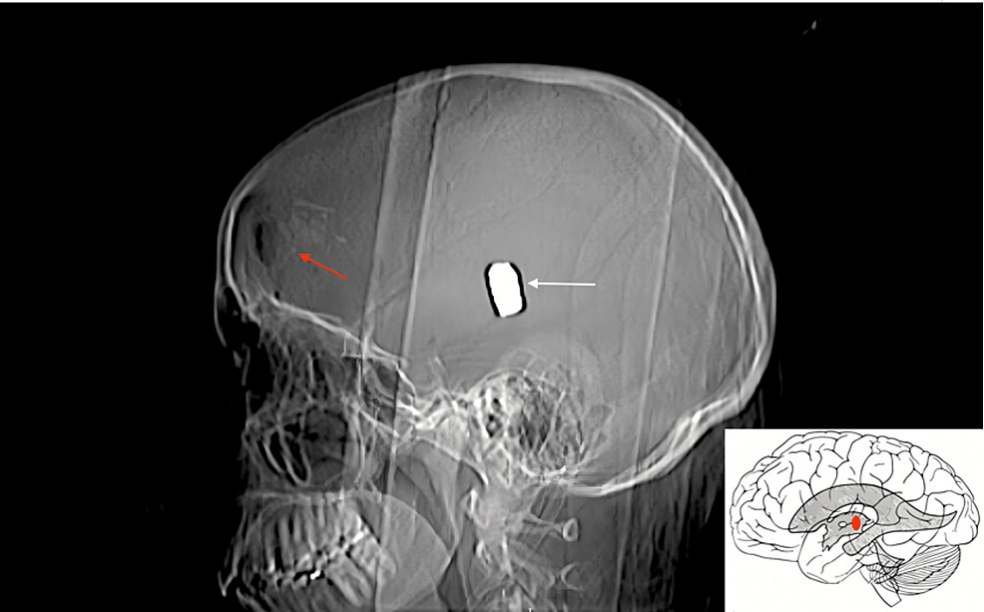
CT head scout image demonstrates the bullet (white arrow) within the third ventricle. Schematic illustration of the bullet lodged in the third ventricle. Red arrow = comminuted full-thickness frontal fracture.

We present a case of a 37-year-old male who presented to the emergency department following a gunshot injury to the head, resulting in the lodgment of the bullet within the third ventricle. The patient arrived hemodynamically stable, with a Glasgow Coma Scale (GCS) score of 14 (E4V4M6). He exhibited intact cranial nerve function and only mild confusion. Initial computed tomography (CT) imaging of the head demonstrated the bullet lodged within the third ventricle (Figure 1). An external ventricular drain (EVD) was placed in the right frontal horn to prevent hydrocephalus and a small frontal craniotomy was performed for debridement of the entry site and dural repair. The patient received cefazolin 2 g IV at the induction of the frontal craniotomy and was kept on cefazolin 2 g IV every eight hours (q8h) for five days. At the time of the first operation, the bullet was located in the third ventricle, so we decided against the removal through an endoscopic approach due to concerns of injuring the fornix during the bullet's retrieval through the foramen of Monro. The EVD was kept open at 15 cm during the preoperative time, with low drainage.

**Figure 2:**
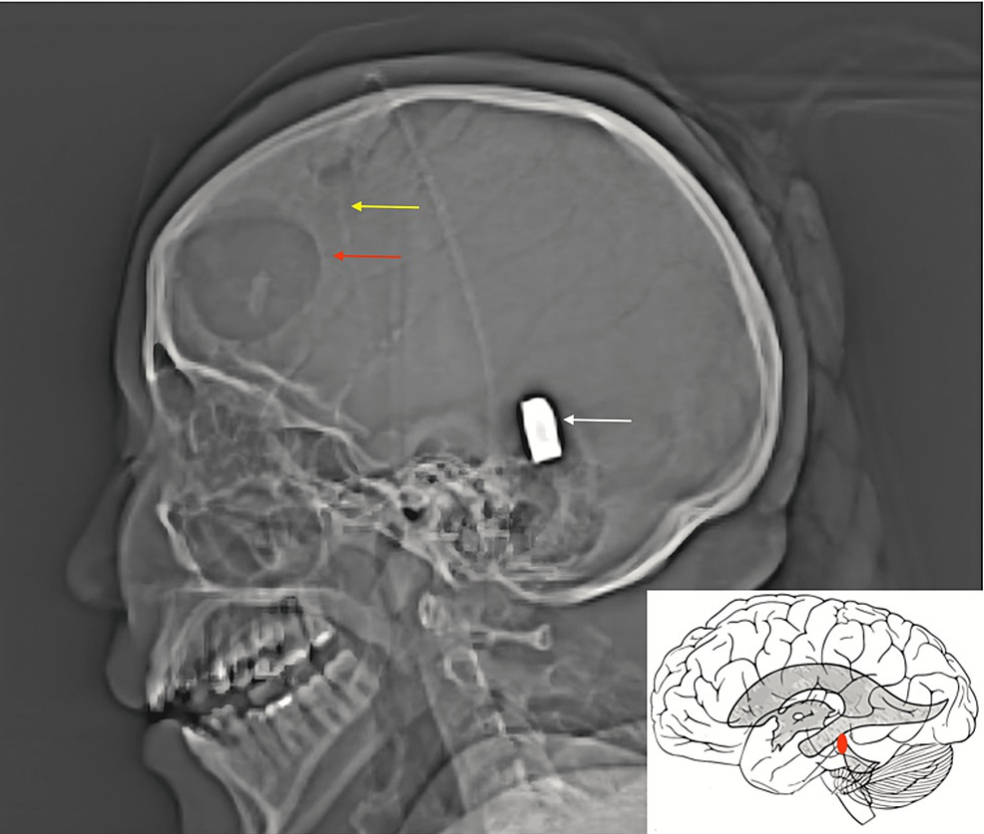
CT head scout image demonstrates the migration of the bullet (white arrow) into the cerebral aqueduct. A small frontal craniotomy (red arrow) and an external ventricular drain into the right frontal horn (yellow arrow) are noted. Schematic illustration of the bullet migrating through the cerebral aqueduct.

**Figure 3:**
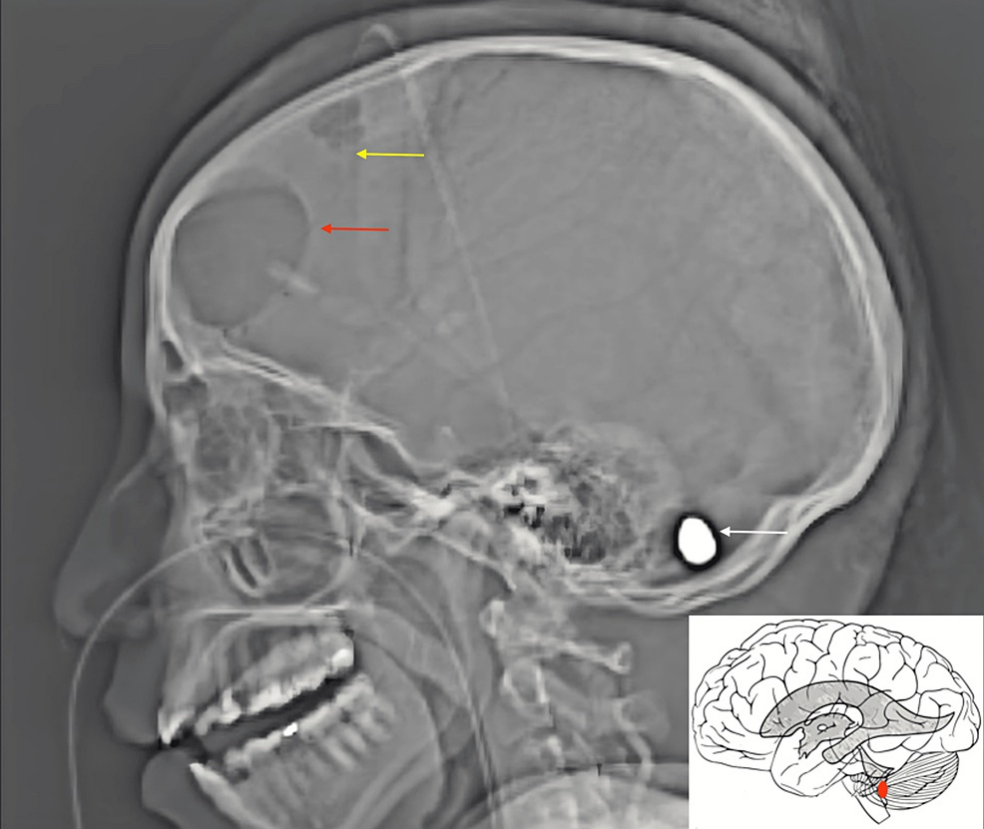
CT head scout image demonstrates the bullet (white arrow) in the posterior fossa, into the fourth ventricle. Schematic illustration of the bullet lodged in the fourth ventricle. Red arrow = frontal craniotomy. Yellow arrow = external ventricular drain.

As we were planning an interhemispheric approach to remove the bullet from within the third ventricle, new CT imaging done after the frontal craniotomy for surgical planning showed that the bullet had migrated, and it was now located at the cerebral aqueduct (Figure 2). The patient was clinically stable and without any new focal deficits during that time, so we decided on repeating neuroimaging in a few hours due to the belief that a surgical approach to retrieve the bullet from within the cerebral aqueduct would carry a high morbidity risk. Repeat CT imaging of the head demonstrated that the bullet had then traversed the cerebral aqueduct reaching the fourth ventricle (Figure 3).​​​​​​​​​​​​​​

**Figure 4:**
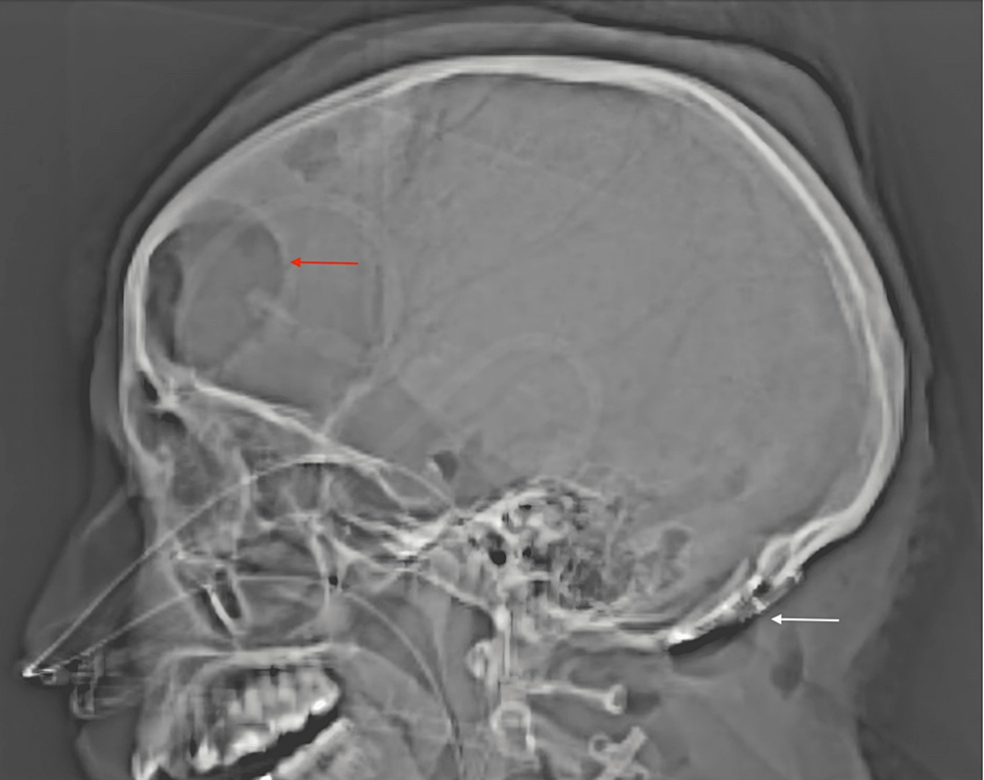
CT head scout image demonstrates the removal of the bullet from the fourth ventricle through a suboccipital craniotomy (white arrow). The external ventricular drain has been removed. Red arrow = frontal craniotomy.

With the bullet now in the fourth ventricle, the patient was taken to the operative room to remove the foreign object. During the positioning of the patient at the OR table and during the whole operative time, the EVD was kept closed. The intracranial pressure was not transduced during the operative time. The patient was positioned prone, with the head flexed, and a suboccipital craniotomy was performed. The distal portion of the bullet was visualized after the initial inspection of the fourth ventricle through the obex. Still, after manipulation of the tonsils for the telovelar approach, the bullet started to "descend" inside the aqueduct. Subsequently, it was not possible to retrieve the bullet using that particular approach. Postoperative imaging three hours after the surgical procedure to monitor the bullet's position revealed its return to the fourth ventricle, prompting a second surgical intervention with the patient in a sitting position. The same craniotomy was used in this approach. Meticulous dissection and careful manipulation were employed to retrieve the bullet from the ventricular system safely. The patient tolerated the procedure without complications. Postoperatively, the patient had an uneventful course and his GCS score was 15 by postoperative day two. The EVD was removed after the retrieval of the bullet (Figure 4).

## Discussion

Intraventricular bullet lodgment following a gunshot head injury is a rare occurrence that poses unique challenges in diagnosis and management. This case report highlights the complexities associated with intraventricular bullet migration and the tailored surgical approach required for successful retrieval.

In 1918, Cushing described a series of 30 cases involving penetrating injuries to the ventricular system, with 16 of those attributed to gunshot head injuries [2]. Among these cases, the foreign objects were successfully retrieved in eight instances. Regrettably, all patients included in this series ultimately succumbed to their injuries. Subsequently, a few reports have emerged regarding the retrieval of foreign objects, primarily projectiles, from different locations within the ventricular system, and different approaches [3-7].

One potential occurrence that can happen after a gunshot wound to the brain is the spontaneous migration of the bullet [7-10]. Previous studies have indicated that this phenomenon can happen in up to 10% of all cases [11-13]. The timeframe for this varies in the literature, but it has been reported to occur within the first 12 hours following the initial trauma [14]. In some instances, like in the present case, the migration occurs within the ventricular system and preempts a complex discussion regarding indications and surgical approaches to retrieve the foreign object. In our case, the concerns for obstructive hydrocephalus and the risk of shunt-dependence, as well as the possibility of transtentorial herniation and direct mass effect from the bullet at the foramen magnum, were important in surgical decision-making. In addition to the bullet migration encountered during the surgical procedure, we encountered another significant challenge pertaining to the retrieval of the foreign object. The metallic composition and rounded shape of the bullet necessitated the use of modified micro instruments. To address this, we employed bayonet forceps with rubber-coated tips, which enabled a secure grip on the bullet. We also considered utilizing a magnet for retrieval; however, its unavailability during the operation restricted its usage in this case.

The movement of intracerebral objects has been associated with the development of abscesses and hematomas [13,15]. Additionally, it has been linked to cerebral softening caused by edema and brain injury [10]. Other factors that have been suggested to contribute to this phenomenon include gravitational forces and the flow of cerebrospinal fluid [10,16]. It is also important to consider these factors when planning the surgical approach. In our case, we were surprised by the fast return of the bullet to the third ventricle after the initial approach in the prone position, and a second approach in a sitting position was required to successfully remove the bullet.

## Conclusions

We report a rare case of intraventricular bullet migration following a gunshot head injury, highlighting the significance of understanding this phenomenon for guiding surgical management. This case report provides insights into the surgical indications and challenges associated with determining the appropriate approach strategy. In particular, surgical positioning is of paramount importance as foreign objects may migrate during the operative time, necessitating the surgeon to adapt their approach accordingly. By comprehensively addressing the surgical considerations in this case, we emphasize the importance of meticulous planning and ongoing adaptability.
